# Drug decriminalization: a co-designed study outlining the implications for providers of youth services

**DOI:** 10.1186/s12954-025-01320-x

**Published:** 2025-10-21

**Authors:** Nicole Morgan, Jennifer Suen, Una Liao, Sarah Adair, Lyn Heinemann, Sylvia Lai, Kirsten Marchand, Skye Pamela Barbic

**Affiliations:** 1 Department of Occupational Science and Occupational Therapy, Faculty of Medicine, University of British Columbia, 317-2194 Health Sciences Mall, Vancouver, BC V6T 1Z3 Canada; 2 Foundry, 915-1045 Howe Street, Vancouver, BC V6Z 2A9 Canada; 3 Providence Health Care, 1081 Burrard Street, Vancouver, BC V6Z 1Y6 Canada; 4 Foundry Vancouver-Granville, 1260 Granville Street, Vancouver, BC V6Z 1M4 Canada; 5 Centre for Advancing Health Outcomes, 588-1081 Burrard Street, Vancouver, BC V6Z 1Y6 Canada

**Keywords:** Adolescents, Young adults, Youth, Toxic drug crisis, Drug decriminalization, Integrated youth services

## Abstract

**Background:**

Death by drug toxicity is now the leading cause of death among youth in British Columbia (BC). In January 2023, BC implemented decriminalization for personal possession (2.5 g) of certain substances for individuals 18 and over. This research aimed to gain a deeper understanding of service providers who work with youth (ages 15–24). Specifically, the study aimed to explore: (1) their attitudes and beliefs regarding drug decriminalization, and (2) the knowledge and resources they need to effectively discuss drug decriminalization with their clients.

**Methods:**

Community-based participatory research and interpretive description were used to co-design an interview guide and recruitment strategy with leaders at a BC integrated youth services initiative. Fifteen semi-structured interviews were conducted in the fall of 2023 (pre-period of the decimalization repeal in BC) with service providers and data were coded using reflexive, inductive semantic thematic analysis.

**Results:**

The thematic analysis revealed that while decriminalization was perceived as a “step in the right direction,” it remains insufficient to address the needs of youth in BC. Service providers expressed a significant disconnect between the policy and practical support required for youth clients. Despite their strong understanding of youth’s needs, providers reported a lack of involvement in the policy development process.

**Conclusion:**

Service providers said that decriminalization is “a step in the right direction, but not enough.” Additional youth-centred policies and services are needed to address the drug toxicity crisis in BC, and service providers and people who use drugs need a seat at the table to inform, design, and implement policies that will impact youth who use drugs.

## Introduction

Canada is the world’s second highest opioid prescribing nation, behind only the United States [1]. While the toxic drug crisis affects all of Canada, one of the most affected provinces is British Columbia (BC) [1]. With increasing deaths due to drug toxicity, BC declared a public health emergency in 2016 [2]. However, the death rate from the unregulated drug supply has increased, despite expansion of harm reduction and treatment services across BC [3, 4]. Since 2016, more than 13,300 individuals have died from the unregulated drug supply [5]. As this public health emergency continues to kill people who use drugs (PWUD), it becomes clearer that high death rates are not addressed by simple solutions [4]. One group of particular concern is youth. Canadian youth ages 15–24 are the fastest growing population requiring hospital care from opioid overdoses [6]. Similarly, death by drug toxicity is now the leading cause of death among youth in BC; for instance, children and youth accounted for 1.3% of deaths due to unregulated drug toxicity between 2016 and 2023 [4].

With these factors considered, the Government of BC requested an exemption from the federal government’s Controlled Drugs and Substances Act to decriminalize the possession of small amounts of unregulated drugs for people over 18 [7]. According to the initial exemption, from January 31, 2023–2026, individuals in possession of less than 2.5 cumulative grams of cocaine, ecstasy, heroin, fentanyl, or morphine cannot be arrested, charged or have their drugs confiscated by law enforcement [7]. Decriminalization has been proposed as a matter of public health, justice, and equity [8]. It has also been described as a way to destigmatize drug use and possession as well as connect people to care where and when they need it [1, 9–12]. Although much literature has focused on unpacking the effects of decriminalization in BC [13–17], limited research has focused on the perceptions of health providers on the policy change, and we are unaware of any literature in the youth health services space.

Traditionally, reaching youth early in their mental health and substance use trajectory has been challenging [18] and youth in BC have been consistently found to face barriers to accessing and sustaining opioid treatment services [19–23]. In BC, an integrated youth services (IYS) initiative, the BC-IYS, has been established to improve access to mental health and substance use services for youth ages 12–24 years [24]. IYS initiatives provide multiple services at a single access point, thereby facilitating collaboration among various team members [25]. It is important to note that BC’s decriminalization policy applies only to adults ages 18 and older. Youth under 18 are excluded from the Health Canada exemption, which presents unique challenges for service providers working with this age group. With the decriminalization of simple possession in BC, it was uncertain what youth who use drugs (YWUD) and their service providers understood about the exemption and how it would impact service delivery, outcomes, and experiences among youth accessing the BC-IYS. This paper focuses specifically on the perspectives of service providers to examine how decriminalization was interpreted and enacted by those who support young people accessing care. Therefore, this study aimed to better understand (1) What are the attitudes and beliefs of service providers at the BC-IYS around drug decriminalization? and (2) What knowledge and tools would service providers like to be equipped with to discuss the topic of decriminalization with youth? The researchers defined service providers as healthcare professionals and front desk staff that support the delivery of IYS at an urban centre where toxic drug deaths have been consistently high.

It is important to note that during the analysis phase of this study, the decriminalization policy in BC was repealed. The researchers acknowledge that this undeniably shapes how the findings in this study are interpreted and applied. Although the policy’s repeal shifts the terrain, the researchers believe the study contributes novel empirical data to a still-emerging body of literature on youth, substance use, and drug policy. The repeal itself underscores the importance of understanding how policies are implemented, communicated, and received on the ground, especially by those working closely with impacted populations.

## Methods

### Study design

The methodology of this qualitative study was rooted in reflexive thematic analysis and the outlines of Thorne and colleagues [26] for interpretative description. The researchers chose interpretative description for its accessibility, theoretical flexibility, and prevalence in healthcare research [27]. Interpretive description was merged with reflexive thematic analysis because these methodologies synergistically highlight the plethora of participant experiences and translate these experiences into a story arch of various realities and meanings [28]. Given that reflexivity is a vital component of interpretative description and reflexive thematic analysis, the researchers JS, NM, and UL tracked their decision making and met regularly to discuss how potential research biases, impressions, and assumptions might impact their interpretation of the data.

Community-based participatory research (CBPR) was woven into all stages of this research. CBPR is a collaborative research approach that aims to improve the health and social equity of communities by striving for equitable knowledge generation among multiple parties and building on the existing strengths of communities [29]. This study was also rooted in co-design, a human-centred way of conducting research that is increasingly adopted within healthcare research due to its focus on improving healthcare experiences and outcomes [30–32]. Taken together, CBPR and co-design prioritize “end users” (i.e., service providers) and iterative co-creation [31, 33] The researchers prioritized service providers by incorporating their feedback into the thematic analyses. The researchers engaged in co-design by iteratively collaborating with BC-IYS leadership and other members of the research team to create the interview guide and analyze themes. Ethics approval was obtained from the University of British Columbia (#H23-02800).

### Setting

The BC-IYS initiative where this study took place has a provincial central office to support backbone operations, virtual services, and 17 physical centres located across BC (with 18 more in development). The initiative’s core services include care for physical and sexual health, mental health, substance use health, peer support, and social services, which are youth- and family-driven to meet the needs of youth [24]. After consultation with BC-IYS leadership, participants were recruited from a BC-IYS centre in downtown Vancouver that provides substance use services to a high number of youth in downtown; Vancouver is a metropolitan city (2021 population: 2,642,825) with a high rate of unregulated drug toxicity deaths [34, 35].

### Recruitment and sampling

The researchers engaged in purposive sampling to recruit participants in the fall of 2023. Participants were recruited with posters displayed at the BC-IYS centre and through word-of-mouth from other staff members. Service providers met the study inclusion criteria if they spoke English, had worked at the centre for at least one month, and could verbally consent to participation. The inclusion criteria were kept broad to account for the widest representation of service providers working at the centre. Various roles were encouraged to participate in the study, such as occupational therapists, social workers, registered nurses, clinic support assistants, nurse practitioners, peer support workers, physicians, and case managers. These professionals were invited to partake because they are all involved in service provision. To maintain confidentiality, each participant chose or was assigned a pseudonym.

### Procedures

Following CBPR and co-design, the initial interview questions were proposed by three BC-IYS staff members in positions of leadership; then, four different staff in leadership positions provided feedback on the initial questions via virtual interviews. The interviewers (NM, JS, UL) utilized this feedback to develop the next iteration of the interview guide. The team also further refined the guide based on the content of the first three participant interviews. Each interview question was created to explore three topics: (1) experience providing substance use services, (2) personal knowledge of recent decriminalization policy, and (3) impact of decriminalization policy on service provision to youth. Some of the interview questions included: “How, if at all, did drug decriminalization affect the substance use care you provide to youth?”, “What lessons regarding decriminalization would you share with service providers who work in this field?”, and “How, if at all, has the topic of decriminalization come up in your day-to-day work?”.

The interviews took place between September-November 2023; thus, data were gathered seven months after decriminalization was implemented. Interested participants were informed about the aim of the study, the procedures, data privacy, and the professional background of the research team. After giving verbal consent, each participant completed a semi-structured interview. With the implementation of the aforementioned research approaches, researchers NM, UL, and JS conducted 15 semi-structured interviews in a participant-centred manner that highlighted participants’ expertise. Participants were each interviewed once. NM, JS, and UL are Occupational Therapy graduate students with experience conducting qualitative interviews and using qualitative research in practice. They are not employees of the BC-IYS initiative and had no prior connection to participants before interviewing them.

Interviews were conducted either by Zoom (*n* = 6) or in-person in a private office (*n* = 9), depending on participant preferences. For in-person and video interviews, the researchers monitored participants’ tone of voice, facial expressions, and body language. Interviews ranged from 15 to 45 min and averaged a total of 26.85 min. Given most interviews happened during the break period of service providers, it was recommended to make sure service providers could have questions before hand to ensure the interviews lasted around 30 min. Interviews were audio-recorded and transcribed verbatim on Zoom. Transcripts were de-identified, and data were stored on a password-protected file in a secure research environment, in accordance with applicable regulations. Researchers SA and SB supervised data generation and provided feedback during data analyses.

### Data analysis

The de-identified transcripts were analyzed in accordance with Braun and Clarke’s [28] reflexive thematic analysis. Before coding, researchers JS and NM familiarized themselves with the data by re-listening to audio-recordings and re-reading transcripts. They used NVivo 14 [36] to organize and code the semantic content of their transcribed data, using inductive thematic analyses. They independently coded two of the richest de-identified transcript data and met once a week for 3 weeks to review quotes that were challenging to code. They coded remaining transcripts independently. This research involved two coders because the researchers completed this qualitative data analysis as part of their graduate studies. Following the initial coding, researchers JS and NM brainstormed preliminary themes by determining common patterns across the data. They shared a file of combined codes with researcher UL, and all three researchers engaged in theme generalization. To ensure their themes represented the data, they reviewed emerging themes at the level of coded data extracts. These researchers met weekly to organize codes into preliminary themes, distilling and amalgamating the strongest supporting quotes for each theme.

To maintain their commitment to co-design, the researchers NM, JS, and UL presented preliminary themes to their BC-IYS leadership partners and integrated their feedback accordingly. Next, they engaged in triangulation by sharing their thematic analyses with three participants and subsequently revising their analyses. Preliminary results were presented to the BC-IYS rehabilitation and research team at the BC-IYS central office.

## Results

### Demographic characteristics

Fifteen interviews were conducted with primary care providers (*n* = 5; nurses, nurse practitioners, physicians) and allied health care providers (*n* = 10; occupational therapists, rehabilitation assistants, social workers, clinic support assistants, case managers). Participants had worked at the BC-IYS initiative for a minimum of two months and maximum of 9.5 years; however, many participants had prior experience in substance use and harm reduction before working at the BC-IYS. Participants spoke about their experiences working with PWUD and the impact of decriminalization on their work with this population; however, it is important to note that participants work with youth, therefore these findings are applicable to YWUD.

The thematic analysis revealed a significant disjunction between the decriminalization policy framework and the actual needs of YWUD in BC. Service providers expressed a robust understanding of the needs of this demographic; however, they reported a lack of involvement in the policy development and inadequate guidance on its practical implementation. During data collection, participants swiftly transitioned to proposing solutions aimed at enhancing support for youth and integrating forthcoming policies into practices that address the needs of young individuals who use drugs.

**Fig. 1:**
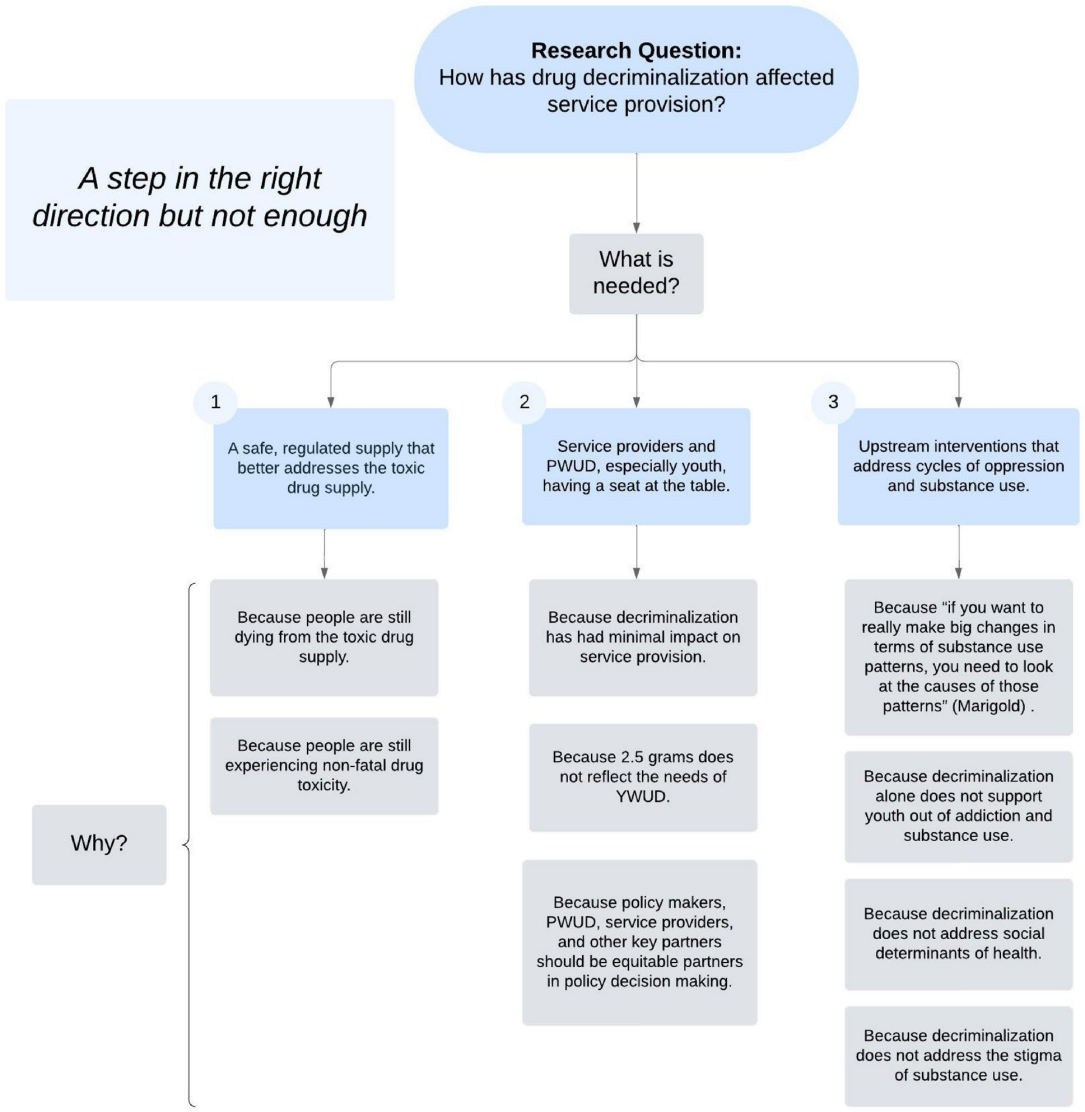
This figure describes the three main themes that were identified. Participants said that BC needs: (1) A safe, regulated supply that better addresses the toxic drug supply (2), Service providers and PWUD, especially youth, having a seat at the table, and (3) Drug policy that happens in a system rather than a vacuum

Figure 1 describes the three main themes that were identified. Participants said that BC needs: [1] A safe, regulated supply that better addresses the toxic drug supply [2], Service providers and PWUD, especially youth, having a seat at the table, and [3] Drug policy that happens in a system rather than a vacuum. The overarching take-away from the findings was that drug decriminalization is a step in the right direction but not enough. Four participants used this language to refer to ways that the decriminalization policy had potential but fell short in its capacity to positively impact the lives of YWUD.

#### Theme 1: a safe, regulated supply that better addresses the toxic drug supply

This theme refers to the attitudes and beliefs of service providers that decriminalization does not go far enough and needs to prioritize increased access to a safe, regulated drug supply to prevent youth death by drug toxicity. As “Taylor” (Registered Nurse) noted, “I definitely think that the government needs to come in and create the safe, regulated drug supply and start saving lives because what we’re doing now just ain’t it.” The BC Centre on Substance Use (2023) defines safe supply as “a legal and regulated supply of psychoactive substances that traditionally have been accessible only through the illicit drug market”. Participants expressed concern that there was a gap between the decriminalization policy and infrastructure needed to provide young people with access to a safe supply.

While some participants were hopeful that decriminalization would reduce criminal justice involvement, they emphasized that both youth and adults continue to be incarcerated and die from the toxic drug supply. Participants said that decriminalization is often misunderstood as a solution to the toxic drug crisis. While they acknowledged that reducing criminalization is important, they stressed that decriminalization does not address the immediate dangers posed by the unregulated, toxic drug supply. As “Bruce” (Social Worker) put it, “The big concern is, is the person I’m seeing going to die tomorrow based on the drugs that are in their pocket right now—whether or not those drugs are criminalized is kind of irrelevant.” Participants emphasized the need for early intervention in youth substance use care but expressed a sense of hopelessness, noting that, without access to a safe supply, their ability to support positive outcomes is severely limited. As “Jane” (Nurse Practitioner) reflected: “I think what we do is, a lot of what we see is very sad, and the reality is just, you know, is decriminalization really gonna change how people are overdosing and dying? I don’t—I’d like to see the stats. I don’t think it’s going to…”.

#### Theme 2: service providers and PWUD, especially youth, having a seat at the table

This theme addresses the implications of not incorporating the perspectives of people who provide and access substance use services into the decriminalization policy. This includes service providers who provide care to youth ages 12–24. Although the Health Canada exemption applied only to adults (18+), most participants noted that the policy impacts the broader ecosystem of youth substance use services. Participants stated that they wished their unique perspectives had been included, as they typically work with young people over age 18 who are transitioning into adulthood. For instance, “Chester” (Addictions Medicine Physician) said, “I feel like, I feel like– I wish I knew the different options that were being discussed at the table because I, you know, I learned all about decriminalization pretty much like as it was being rolled out and all the decisions had been made.”

All participants noted that drug decriminalization had minimal impact on their substance use services. “Chester” noted, “I don’t think it’s really changed how I practice. I think I’ve practiced more or less the same way before and after.” Similarly, “Karmel” (Registered Nurse) said, “There hasn’t really been a huge shift in the way we’ve provided care because that’s always the lens we’ve looked at it through.” Participants also noted that drug decriminalization was not coming up in their conversations with youth and families; as a result, they felt that they did not require additional training or tools to navigate conversations around decriminalization. For instance, “Taylor” (Registered Nurse) said:

It hasn’t at all – changed anything that I do. Because I think again, I will say, like the people that are in it, like working frontline in it like my colleagues that are in the Downtown Eastside [an area of Vancouver with a high number of street-entrenched individuals who experience barriers to participation in the community, such as substance use] and my colleagues here, I think we all very much understand that just decriminalizing doesn’t do anything for supporting the youth out of addiction.

Participants highlighted a disconnect between the policy’s design and the realities faced by YWUD, particularly in relation to the 2.5-g possession limit. Six participants shared that the youth they work with frequently carry and use more than 2.5 g in a single sitting. Participants also agreed that the threshold does not align with the purchasing or consumption patterns of all YWUD. As “Taylor” (Registered Nurse) stated, “2.5 is a joke. I’ve got clients that do that in a single shot in a day. How is this helpful?”

While critiques of the 2.5-g threshold have been raised in other studies [16, 37], the youth-serving providers of this study emphasized the importance of having their unique perspective understood through a public health lens [15]. Participants emphasized that the limit fails to account for young people who may be sourcing drugs for multiple days. Participants raised serious concerns about continued criminalization and safety risks, even within a supposedly decriminalized context. As Taylor put it, “The goal should be ending overdose deaths, right? And 2.5 g—like, limiting an amount—doesn’t stop the poisoned drug supply. That’s the problem: the poisoned supply, not how much someone has.”

Finally, participants emphasized the urgent need for equitable and meaningful partnership in policymaking—one that includes YWUD, their families/caregivers, service providers, and other key stakeholders rather than giving inequitable decision-making power to organizations such as law enforcement. For instance, Taylor said “[The police] had a lot of influence in that and they actually went against what all of the coalitions, the [drug users support network] were saying like [drug users compassion club advocating for safe supply] and other programs, even health programs, are suggesting a much higher dose but the [local police force] had far too much influence. But that’s because we have a militarization of healthcare.” While some PWUD were involved in shaping BC’s decriminalization policies, one participant felt that youth-specific perspectives (especially transition age youth) were largely overlooked and four participants noted that the recommendations of service providers were not meaningfully incorporated. Critically, they pointed to a lack of planning for implementation across the broader health system, particularly in areas such as youth substance use services and integrated youth services such as the BC-IYS. Participants expressed frustration at working within a system that promotes upstream, youth-centred care, yet excludes youth and those who support them from policy decisions that directly impact their lives. Without involving service providers in every step of policy creation, participants noted that policies risk being ineffective in addressing the urgent needs of young people navigating substance use in BC.

#### Theme 3: drug policy that happens in a system rather than a vacuum

The third theme highlights participants’ belief that while decriminalization may reduce some harms associated with criminal justice involvement, it falls far short of addressing the deeper, structural drivers of substance use among youth. Participants emphasized that decriminalization alone does not confront the social determinants of health, such as poverty, unstable housing, systemic racism, and barriers to education and employment, that shape drug use and related harms in the first place. Five participants said that additional policies are needed to address the social determinants of health that can contribute to poor health outcomes among youth and adults who use drugs, such as social inclusion and non-discrimination, early childhood experiences, and access to housing, education, employment, and income.

Participants stressed the need for upstream, prevention-focused policies that respond to the root causes of substance use. For example, “Marigold” (Occupational Therapist) reflected:

The intervention—including decriminalization—needs to happen way upstream. People who end up deeply entrenched in substance use are often those failed repeatedly by systems: family, health care, education, justice, foster care. And sometimes, even just one of those failures—like childhood abuse—can lead someone down this path. If we really want to make an impact, we need to invest in prevention much earlier.

Participants called for policy approaches that go beyond individual behavior change and address systemic inequality. “Taylor” (Nurse) explained:

Housing is a great way to support people out of substance use—safe, dignified housing, or access to education. The social determinants of health are what actually support people out of addiction and away from death. And this policy doesn’t touch any of that.

Echoing this, Marigold added:

The system just isn’t set up to help people succeed. If we want to change substance use patterns, we need to look at what causes those patterns and invest accordingly. It’s really not that radical.

Finally, participants noted that decriminalization has done little to reduce the stigma of drug use and possession. While the policy aimed to promote destigmatization [38], no participants reported seeing real change in attitudes. “Glen” (Occupational Therapist) observed:

If someone already holds stigma around drug use, that’s not going to disappear just because it’s been decriminalized.

Similarly, “Bruce” (Social Worker) shared skepticism about how quickly change might come:

If we wanted to see a real shift in stigma, that’s years and years away. I’ll be lucky to see it in my lifetime.

Together, participants’ insights indicate the limitations of decriminalization when implemented in isolation. Without addressing the broader social conditions that shape substance use—and without sustained efforts to challenge stigma—the impact of decriminalization on the health and wellbeing of youth will remain limited.

## Discussion

Government policies are instrumental in shaping the healthcare landscape, directly influencing the provision and accessibility of clinical care for patients, including youth. In light of BC’s drug decriminalization, this study aimed to explore the impact of decriminalization on service providers delivering care to youth in BC. The findings revealed a significant disconnect between service providers and policy implementation. Specifically, service providers expressed feelings of exclusion from both the decision-making process and implementation of the policy. While they acknowledged the policy as a positive step forward, they regarded it as insufficient in its current form. These findings are consistent with other research which suggests that the success of decriminalization is maximized when implemented with additional policies [9, 14, 17, 39].

As noted in the introduction, the decriminalization policy has undergone revisions since the commencement of this research, trending toward a re-criminalization approach. Despite these developments, the findings of this study retain their relevance for future policymakers and service providers involved in substance use care for youth and families in BC and beyond. The insights generated offer valuable lessons about the policy implementation process, notable gaps in communication, and practical challenges that arose in real time. This research highlights how insights can inform future iterations of drug policy, especially in contexts where decriminalization may be reconsidered or implemented differently.

The themes of this study align with Duong’s perspective [40] that, while many British Columbians support decriminalization, its potential benefits are compromised by several missing components. They note that the success of decriminalization depends on whether service providers are able to provide PWUD with alternatives and divert them to other services [40]. Duong, as well as Bonn and colleagues [41], argue that decriminalization must be implemented alongside access to social supports and safe, regulated drugs. Furthermore, a report to the Chief Coroner of BC suggests that unregulated toxic drugs are driving the current drug toxicity emergency and that increased access to safe, regulated drug supply is needed to save lives [4]. The finding that service providers and PWUD need a seat at the table is supported by other researchers’ findings that the policy’s 2.5-g exemption is too low and not conducive to the buying practices of many PWUD [40].

Although service providers and advocates called for a higher exempted amount [13], the amount was set at 2.5 g due to pressure from law enforcement [14, 16]. Early research in BC suggests that low thresholds have not been impactful [42]. Consequently, participants felt that decriminalization did not protect PWUD who are most at risk of poor health outcomes related to substance use, such as youth. For instance, street-entrenched individuals who buy drugs in bulk need to carry larger amounts on their person due to not having a secure storage [40]. Insights from Portugal’s drug decriminalization underscore the importance of establishing objective, substance-specific limits that align with people’s needs [42]. Service providers can play a pivotal role in collaborating with youth to clarify these established limits, offer psychoeducation regarding consumption levels, and deliver pertinent harm reduction support and treatment.

The importance of upstream interventions that address cycles of oppression is consistent with the findings of existing research that the social determinants of health, such as income, unemployment, and housing, are highly associated with the health outcomes of people who use opioids [43]. This research, in the youth context, articulates that health policies need to address the social determinants of health to effectively support the needs of YWUD [40]. Participants in the current study noted that these health policies are missing from BC’s response to the toxic drug crisis.

Canadian youth ages 15–24 are the fastest-growing demographic requiring hospitalization for opioid use [44], yet BC’s decriminalization policy excludes YWUD under 18. Participants noted that this exclusion contradicts literature indicating that youth face increased risks of death and non-fatal drug toxicity, particularly given their existing barriers to opioid treatment, due to a lack of access to a safe, regulated drug supply [20, 21]. Therefore, policies must include and protect youth in BC, with upstream interventions having significant impacts on YWUD. Participants also stressed that current policies often overlook youth’s needs by excluding them from co-design processes and failing to create developmentally and culturally relevant implementation strategies. The findings suggest that service providers who work with youth are uniquely positioned to advocate for the needs of youth and ensure the representation of youth perspectives in policy decision making. Professionals with clinical experience in substance use, especially with youth, have the capacity to influence policy and advocate for the inclusion of individuals with lived experiences in the policy-making process. As the re/decriminalization policy in BC evolves [39], meaningful, evidence-based, and co-designed policies are crucial to prevent mortality and empower individuals to continue living in a meaningful way. The current study highlights the importance of maximizing collaboration in policy development to improve health outcomes for all community members.

### Limitations

This study has several limitations. First, this study relies on the personal experiences and perspectives of service providers working in a single urban IYS setting. These service providers bring their own positionality to the research interviews, and these positions may be influenced by service providers’ social locations, their professional training and roles, and their personal values and experiences. Therefore, the findings may not directly transfer to service providers working in other health care settings (e.g., emergency departments). Second, the research was conducted in a single site in urban Vancouver, a setting characterized by a high prevalence of substance use, robust harm reduction infrastructure, and comparatively progressive drug policies. Thus, these contextual factors may not reflect conditions in rural, remote, or lower-prevalence regions where access to services and community norms around drug use may differ significantly. As such, the transferability of the findings to other geographically diverse settings may be limited. Future research should explore the implementation and impact of decriminalization in a broader range of geographic and service contexts. Third, the exclusion of youth under 18 from BC’s decriminalization policy represents a key limitation. This policy gap poses distinct challenges for youth-serving providers and leaves unanswered questions about how decriminalization and other substance use policies affect younger populations. Fourth, the shorter duration of some interviews may have impacted the researchers’ ability to establish rapport and foster participants’ comfort to provide answers that highlighted their personal perspectives and beliefs on decriminalization. Finally, given the evolving nature of drug policy in BC, including the repeal of decriminalization, the relevance and applicability of this study’s findings may shift over time. Ongoing research is needed to understand how policy changes continue to shape the experiences of youth, service providers, and communities.

## Conclusion

To the researchers’ knowledge, this is one of the first known studies to strategically combine the complementary features of interpretive description, CBPR, and co-design to understand the impact of drug decriminalization on service provision at an IYS centre. The qualitative findings revealed that while drug decriminalization is a step in the right direction, it is not enough to support the needs of YWUD. Service providers highlighted that additional policies and social services are needed to support this population effectively. Although drug decriminalization aims to reduce the stigma of drug use and encourage access to life-saving services, the findings suggest that stigma persists, contributing to ongoing fatalities and incidents of drug toxicity. The service providers indicated that, in addition to decriminalization, BC requires a safe, regulated drug supply, active involvement of service providers and PWUD in policy decision-making, and drug policy that happens in a system rather than a vacuum. Addressing the limitations of drug decriminalization and proposing viable integrated solutions is crucial for the health and well-being of YWUD.

## Data Availability

The dataset generated during and/or analyzed during the study are not publicly available due to pseudo anonymity of research participants, but are available from the corresponding author on reasonable request.
